# Measuring attitudes toward communication skills in nursing and health sciences: psychometric properties of the CSAS in Spain

**DOI:** 10.3389/fpubh.2026.1879823

**Published:** 2026-07-13

**Authors:** Laura Cubero-Plazas, Jesús Privado, David Sancho-Cantus, Elena Castellano-Rioja, Cristina Cunha-Pérez, Montserrat Cañabate-Ros

**Affiliations:** 1 Department of Nursing, Catholic University of Valencia, Valencia, Spain; 2 Nursing and Mental Health Research Group, Department of Nursing, Catholic University of Valencia, Valencia, Spain; 3 Department of Psychobiology and Methodology of Behavioral Sciences, Universidad Complutense de Madrid, Madrid, Spain; 4 Hospital Psychiatry Unit, Hospital Peset of Valencia, Valencia, Spain

**Keywords:** communication skills, CSAs, health sciences, interpersonal communication, soft skill competencies

## Abstract

**Introduction:**

The communication skills of healthcare professionals directly influence interventions with patients and their families, and therefore, the quality of care provided.

**Aim:**

To adapt and validate the CSAS questionnaire in a Spanish university sample of Health Sciences students: Nursing, Psychology, Physiotherapy, Medicine, among others.

**Materials and methods:**

A cross-sectional design was employed with a sample of 792 participants (21.9% male) with a mean age of 21.12 years. The internal structure, internal consistency, factorial invariance, differential validity by sex, and convergent and discriminant validity of the instrument were analyzed. Normative data were also calculated.

**Results:**

Two factors (positive and negative attitudes) with a correlation of −0.71 were obtained, demonstrating adequate fit (GFI = 0.967, NFI = 0.920, SRMR = 0.059) and with an internal consistency of 0.852 and 0.632, respectively. The factorial structure showed clear differences by sex, with different correlations between both factors: −0.77 for males and −0.68 for females. The test demonstrated adequate convergent validity with other similar measures. Differences in scores were observed by sex, with higher values on the scale for males.

**Conclusion:**

The Spanish version of the CSAS is a valid instrument for evaluating interpersonal communication skills, both generally and specifically in the field of Health Sciences. The establishment of normative data for the CSAS will allow for the identification of the relative position of evaluated individuals in relation to a university sample.

## 1 Introduction

Effective communication skills (CS) are indispensable for any interaction between healthcare professionals and patients. In this context, it is critical that healthcare providers not only assess patients’ care needs but, more importantly, listen attentively and understand their emotions (1).

Among the core communication skills, active listening, emotional validation, empathy, and trust constitute the foundation of what is referred to as the therapeutic relationship (2–4). Furthermore, factors related to both the healthcare provider and the patient—such as insecurity, stress, fear, or pain—can significantly impede communication, rendering it ineffective and severely impacting the patient and the quality of care provided (5, 6).

CS are generally presumed to be an integral component of health sciences education. However, it remains difficult to pinpoint exactly when these skills are explicitly addressed during academic training and whether they are thoroughly developed (7, 8). In this regard, studies such as the one by Ferrández-Anton et al. (9), highlight the limited inclusion of CS content within the curricula of university health programs, such as Psychology, Nursing, and Medicine. Health sciences education emphasizes that patient care is centered around the individual. The concept of patient-centred care involves incorporating the patient’s perspective, which facilitates decision-making and treatment adherence, ultimately strengthening the therapeutic relationship (10–12).

In clinical settings, there are various scales designed to assess communication skills. However, these tools often have limitations, such as being restricted to specific nursing contexts or lacking translation and validation for broader application (13).

The *Self-Efficacy Questionnaire* (SE-12), developed by Axboe et al. (14), assesses self-efficacy within the context of clinical communication skills training through 12 Likert-type items, all grouped under a single factor representing general clinical communication competencies. The instrument demonstrates high internal consistency, with a Cronbach’s alpha (*α*) of 0.95. Subsequent studies have reported slightly lower internal consistency values of 0.71 and a test–retest reliability of 0.71 (with a range from 0.63 to 0.75). In terms of content validity, there was strong agreement regarding the relevance of the questionnaire items. Exploratory factor analysis revealed that the 12 items load onto a single factor, which is indicative of self-efficacy.

The *Empathy Management Scale* (EMS) developed by Mora-Pelegrín et al. (15) assesses the processes involved in managing empathy. The scale comprises 18 items organized into five dimensions: identification, incorporation, reverberation, separation, and projection. It articulates empathy processes based on both cognitive and emotional dimensions. This instrument is particularly relevant for healthcare professions where empathy is critical for fostering positive interactions with patients. In terms of evidence for internal structural validity, exploratory factor analysis identified five factors aligned with the proposed dimensions, accounting for 62.4% of the total variance. The internal consistency for the various dimensions yielded Cronbach’s alpha (*α*) values ranging from 0.72 to 0.79.

The *Therapeutic Communication Scale* (TCS), developed by Han et al. (16), evaluates the therapeutic communication competencies of nursing students. It consists of 15 Likert-type items. Confirmatory factor analysis revealed two factors: relationship building and problem-solving. Regarding criterion validity, the TCS demonstrated a moderate correlation with other assessments measuring similar constructs, such as self-efficacy (r = 0.64). The internal consistency values ranged from 0.81 to 0.89, while the test–retest reliability was established at 0.96.

The *Professional Nursing Communication Competence* scale (IMC-CPE), formulated by Soares et al. (17), assesses nursing communication competence through 46 Likert-type items encompassing three dimensions: knowledge, skill, and attitude. Regarding content validity, the level of agreement among experts was 80%. However, the authors did not provide values for the internal consistency of the instrument.

The *Health Professionals Communication Skills Scale* (HP-CSS), validated by Leal-Costa et al. (18, 19), evaluates communication skills across various healthcare professionals. It consists of 18 Likert-type items organized into four dimensions: informative communication, empathy, respect, and social skills. The analysis of the internal structure was conducted using a four-factor oblique model based on the original scale, which demonstrated a need for improved fit. The questionnaire exhibited good internal consistency (*α* = 0.88) for the overall scale and for its dimensions, which ranged from 0.70 to 0.77.

Finally, the *Communication Skills Attitude Scale* (CSAS) created by Rees et al. (20), explores the attitudes of health sciences students within the context of learning and teaching communication skills. It consists of 26 items divided into two subscales: Positive Attitudes (PAS) and Negative Attitudes (NAS). The scale assesses students’ perceptions regarding the manner in which communication skills are taught, the importance of possessing effective communication skills for passing examinations and becoming proficient physicians or nurses, and the utility of these skills in demonstrating respect for both patients and peers (21). The internal consistency of the two subscales was found to be high, with a Cronbach’s alpha of 0.87 for PAS and 0.81 for NAS among undergraduate medical students. In a subsequent study utilizing this scale with a population of medical students, gender differences were identified, with male students displaying higher scores in PAS and lower scores in NAS (22).

The CSAS is among the most frequently utilized instruments for evaluating communication skills among healthcare professionals and has been adapted into several languages, thereby highlighting the significance of the present study (23–25). Since its inception, the CSAS has undergone extensive validation efforts to establish its reliability and applicability across diverse contexts and populations (Table 1).

**Table 1:** Validations of the CSAS.

Language	Author/s	Sample	Dimensions	Reliability
Norwegian	Anvik et al. (56)	1,833 medical students (63.2% female)	Two factors [Positive Attitudes (PAS), Negative Attitudes (NAS)], three dimensions: learning, importance, and respect.	Learning: α = 0.861 Importance: α = 0.532 Respect: α = 0.775
Turkish	Harlak et al. (28)	179 medical students (54% female)	Two factors: Positive Attitudes (PAS) and Negative Attitudes (NAS)	α = 0.65 (NAS) α = 0.90 (PAS)
Korean	Ahn et al. (23)	582 medical students (32.8% female)	Two factors, five dimensions: facilitating interpersonal skills, importance within a medical context, motivation, assessment, and overconfidence.	α = 0.752 (facilitating interpersonal skills) α = 0.744 (importance within a medical context) α = 0.680 (motivation) α = 0.446 (assessment) α = 0.496 (overconfidence)
Catalan	Molinuevo et al. (21)	569 medical and nursing students (72% female)	Two factors: Positive Attitudes (PAS) and Negative Attitudes (NAS)	α = 0.64 (NAS) α = 0.83 (PAS)
German	Busch et al. (26)	529 medical students (64% female)	Two factors: Positive Attitudes (PAS) and Negative Attitudes (NAS)	α = 0.838 (NAS) α = 0.864 (PAS)
Iranian	Yakhforoshha et al. (58)	410 medical students (52.7% female)	Two factors: Positive Attitudes (PAS) and Negative Attitudes (NAS), four dimensions: importance of the medical context, excuse, learning, and overconfidence.	α = 0.87 (importance of the medical context) α = 0.67 (overconfidence) Global ICC of CSAS = 0.81
Italian	Ferrari et al. (27)	206 nursing students (48.55% female)	Two factors: Positive Attitudes (PAS) and Negative Attitudes (NAS)	CVI = 96.9%, α = 0.86.
Chinese	Zhang et al. (55)	492 medical students (57.5% female)	Two factors: Positive Attitudes (PAS) and Negative Attitudes (NAS), four factors: importance of communication tools, negative beliefs, motivation, and assessment.	α = 0.771 (importance of communication tools) α = 0.60 (negative beliefs) α = 0.637 (motivation) α = 0.704 (assessment)
Polish	Panczyk et al. (30)	2,014 nursing students (87% female)	Two factors: Positive Attitudes (PAS) and Negative Attitudes (NAS)	α = 0.700–0.901 (PAS) α > 0.802 (NAS)
Malay	Mohamad-Isa et al. (29)	218 medical students (72% female)	Two factors: Positive Attitudes (PAS) and Negative Attitudes (PAS)	α = 0.815 (PAS) α = 0.614 (NAS)

The majority of validations recognize the two factors proposed by the original scale: Positive Attitudes (PAS) and Negative Attitudes (NAS) (21, 26–30). The PAS subscale demonstrates greater internal consistency, with Cronbach’s alpha (*α*) values ranging from 0.70 to 0.90, in contrast to the NAS subscale, which exhibits values between 0.61 and 0.84. All validations include samples of nursing and medical students, with a higher proportion of female participants. Some studies, consistent with the original validation, indicate that women exhibit more positive attitudes (21, 26); whereas others report no significant differences in PAS and NAS based on gender (27).

In terms of the sociodemographic variables and their impact on CS, age and gender are essential factors to consider when assessing the acquisition of CS during academic training. Santos et al. (31), identify significant differences associated with age—specifically in the dimensions of environmental control and assertiveness—and gender, particularly in the dimensions of environmental control and availability, indicating that female participants tend to achieve higher scores. Gutiérrez-Puertas et al. (32), report, conversely, that males achieve higher scores in communication skills. Ezeala et al. (33), find that women demonstrate a more favourable attitude toward learning CS. Furthermore, studies indicate that women are more likely to pursue professions that require elevated levels of communication skills (34); and typically perform better in CS and collaboration, attaining higher scores in receptive learning strategies and research-based learning (35). Additionally, Busch et al. (26), observe that women score lower on the Negative Attitudes subscale while achieving higher scores on the Positive Attitudes subscale.

The exploration of gender differences in communication skills attitudes remains a complex and often contradictory domain within health sciences education literature. While a predominant body of research traditionally reports that female students exhibit significantly more positive attitudes (PAS) and lower negative attitudes (NAS) due to gendered socialization patterns that emphasize empathy and relational skills, counter-intuitive findings—where male students demonstrate equal or higher valuation of communication training—warrant a deeper multi-faceted examination (36–38).

From a cultural and social expectations perspective, traditional gender stereotypes have historically categorized communication and emotional expression as ‘communal’ or feminine traits, leading some male students in older cohorts to dismiss communication training as a ‘soft’ or non-essential science. However, contemporary shifts in medical and healthcare curricula have increasingly made explicit interpersonal skills as core clinical competencies tied directly to diagnostic accuracy and patient safety. Consequently, modern social expectations within professional academic environments may encourage male students to consciously over-value these skills, recognizing them as technical tools required for professional status rather than merely intuitive traits. Furthermore, potential methodological artifacts and response biases must be considered when evaluating these disparate outcomes. Self-report instruments like the CSAS are inherently susceptible to social desirability bias. Female students, who are often assumed to naturally possess high communication competence, might respond to the scale with less compensatory inflation. Conversely, male students, aware of the academic and institutional emphasis on humanistic care, might exhibit a stronger acquiescence bias or social desirability response, explicitly inflating their self-reported attitudes to align with modern institutional rubrics. Additionally, the highly skewed sex ratios typical in health sciences samples (often predominantly female) can introduce significant sampling variance, potentially leading to statistical artifacts where a small, highly motivated subgroup of male participants inflates the male mean scores (39–41).

Communication skills are intricately linked with other constructs, such as effective communication, empathy, respect, and social skills, across various interpersonal contexts and situations. Reith-Hall and Montgomery (42) delineate dimensions within communication skills, including informative communication, empathy, respect, and social skills, which warrant evaluation in conjunction with other relevant aspects of communication and social competencies. Consequently, it is reasonable to expect correlations among these constructs (43). Furthermore, social skills—defined as the ability to engage effectively with others in diverse social contexts—empathy, which is critical for the establishment of robust interpersonal relationships, and respect, which contributes to fostering a respectful and collaborative communication environment, are essential components of communication skills (19).

Besides, factors associated with the awareness of one’s own emotions and those of others, along with emotional self-regulation, are fundamental to the development of effective communication and social skills. Effective communication and healthy interpersonal relationships are fundamentally contingent upon emotional expression and the capacity to manage emotions in problem-solving contexts (44).

Furthermore, various aspects related to social skills, such as engaging with unfamiliar individuals, expressing positive emotions, and responding to criticism, are prevalent in everyday interactions and necessitate appropriate communication and social competencies. Essential skills for fostering healthy and functional relationships across diverse social contexts include the ability to remain composed in challenging situations, public speaking, advocating for one’s rights, offering apologies, and declining requests (45). Collectively, these competencies contribute to the establishment and maintenance of positive interpersonal dynamics.

Considering this, the primary objective of this study was to adapt and validate the Communication Skills Attitude Scale (CSAS) within a Spanish sample of health science students. This demographic is of particular interest due to the critical role that communication plays in the healthcare context and its significance in patient interactions (46). Furthermore, other objectives were to analyze the internal structure of the scale to determine its dimensions and their interrelationships, as well as to calculate the internal consistency of each identified factor. The research aimed to assess evidence of factorial invariance based on gender to ensure structural consistency across sexes, while examining the relationship between scale dimensions and participant age. Additionally, the study sought to obtain evidence of convergent and discriminant validity by relating the CSAS to measures of social and communication skills and emotional intelligence, while simultaneously investigating differential validity by gender and establishing reference values for the evaluated sample.

## 2 Methods

### 2.1 Participants

The sample consisted of 792 participants, with a mean age of 21.12 years (SD = 4.70 years), of whom 21.8% were male. Among these individuals, 26.5% were non-university students, while the remainder were university students in their first to fourth years. The fields of study were as follows: 65.2% were enrolled in Nursing, 5.8% in Psychology, 5.3% in Physiotherapy, 7.3% in Medicine, 5.4% in Nutrition, 3.0% in Podiatry, and the remaining participants were pursuing other academic disciplines.

### 2.2 Instruments

The *Communication Skills Attitude Scale* (CSAS), developed by Rees et al. (20), evaluates the attitudes of health science students within a learning and teaching context through 26 items employing a five-point Likert scale. This instrument comprises two subscales: Positive Attitudes (PAS) and Negative Attitudes (NAS).

The *Trait Meta-Mood Scale* (TMMS-24) (47) is a self-report measure of emotional intelligence, consisting of 24 items assessed on a five-point Likert scale, which are organized into three subscales: Emotional Attention, Emotional Clarity, and Emotional Repair. Table 2 provides the reliability metrics for all instruments utilized in the study.

**Table 2:** Descriptive statistics, internal consistency (Cronbach’s *α*), and internal discrimination (corrected item-total correlation) for the measures.

Measure	*M*	*SD*	Skewness	Kurtosis	α	Corrected item-total correlation
Emotional attention (TMMS)	30.32	6.00	−0.58	0.08	0.875	
Emotional clarity (TMMS)	27.89	5.91	−0.20	−0.32	0.877	
Emotional repair (TMMS)	28.65	5.68	−0.15	−0.34	0.829	
Emotional intelligence (TMMS)	86.86	12.16	−0.23	0.13	0.864	
Positive attitudes (CSAS)	56.06	5.76	−0.64	−0.40	0.852	0.42 to 0.61
Negative attitudes (CSAS)	27.15	5.70	0.28	−0.31	0.632	0.22 to 0.47
Interacting with strangers (CHASO)	13.31	3.62	−0.15	−0.43	0.800	
Positive feelings (CHASO)	17.45	2.93	−1.31	1.47	0.817	
Coping with criticism (CHASO)	15.82	3.12	−0.53	0.00	0.829	
Interacting with acquaintances (CHASO)	11.61	4.76	0.03	−0.96	0.925	
Calmness in the face of criticism (CHASO)	14.27	3.13	−0.34	0.10	0.735	
Public speaking/interacting with superiors (CHASO)	13.26	3.91	−0.16	−0.65	0.824	
Coping with ridicule (CHASO)	12.13	3.42	−0.10	−0.13	0.660	
Defending rights (CHASO)	14.69	3.51	−0.47	−0.10	0.773	
Apologizing (CHASO)	17.88	2.32	−1.11	0.93	0.761	
Refusing requests (CHASO)	14.87	3.39	−0.41	−0.33	0.785	
Total SOCIAL Skills (CHASO)	145.28	20.98	−0.23	0.14	0.808	
Empathy (EHC-PS)	23.15	2.35	−1.56	2.40	0.823	
Informative communication (EHC-PS)	26.66	2.89	−0.95	0.56	0.701	
Respect (EHC-PS)	14.14	1.33	−1.71	2.69	0.742	
Social skill (EHC-PS)	15.25	2.53	−0.12	−0.47	0.463	
Total communication skills (EHC-PS)	83.79	7.79	−1.04	1.21	0.867	

M, mean; SD, standard deviation.

The *Health Professionals Communication Skills Scale* (EHC-PS) (48) comprises 18 items rated using a five-point Likert scale, assessing four dimensions: Informative Communication, Empathy, Respect, and Social Skills.

*The Social Skills Questionnaire (CHASO)* (45) consists of 40 items rated on a five-point Likert scale, evaluating 10 specific skills: (1) Interacting with individuals of interest, (2) Advocating for one’s rights, (3) Public speaking and interacting with authority figures, (4) Maintaining composure in embarrassing situations, (5) Apologizing, (6) Engaging with strangers, (7) Expressing positive emotions, (8) Coping with potentially embarrassing situations, (9) Refusing requests, and (10) Managing criticism.

### 2.3 Procedure

The scale was adapted into Spanish using Brislin’s back-translation methodology (49). Initially, two independent bilingual experts (in both English and Spanish) translated the scale into Spanish. Subsequently, another two bilingual translators translated this version back into the original language of the scale. Potential discrepancies between the original and newly translated versions of the questionnaire were examined to finalize the instrument.

The questionnaires were administered in person, under the supervision of an evaluator, to groups of approximately 50 participants, and were completed using Google Forms. All procedures involving human participants were conducted in accordance with the Declaration of Helsinki. Participants provided written informed consent for their involvement in the study, and they were informed that participation was voluntary and anonymous, with no financial compensation provided. This research received ethical approval from the Research Ethics Committee of the Catholic University of Valencia (UCV/2022–2023/033).

### 2.4 Statistical analysis

First, the distribution of the applied measures and their internal consistency indices (Cronbach’s *α*) were analyzed. Although the 5-point Likert scale is inherently ordinal, responses were modeled as continuous based on the theoretical assumption that respondents conceptualize the items as a continuum during the cognitive process of responding. Second, the factorial structure of the scale was examined through confirmatory factor analysis (CFA). Three types of goodness-of-fit indices were employed to assess the model fit: (1) Absolute indices to evaluate whether the theoretical model fits the empirical data: the *χ*^2^*/df* index (50), where values below 3 indicate a good fit; the Goodness-of-Fit Index (GFI) (51), with values > 0.95 considered acceptable; and the Standardized Root Mean Square Residual (SRMR) (52), where values < 0.08 suggest a good fit (53). (2) Incremental indices, which compare the obtained model to the null model, include the Normed Fit Index (NFI) (50), where values greater than 0.95 indicate a good fit. Additionally, (3) parsimonious indices, which penalize the number of estimated parameters, include the Parsimony Goodness-of-Fit Index (PGFI) (51) and the Parsimony Normed Fit Index (PNFI) (54), both of which are considered indicative of a good fit when values exceed 0.50. Byrne (55) recommends a minimum of 10 participants per indicator for factorial analyses, and in the present study, we included 792 participants and 26 indicators in the most complex model: 792/26 = 30.46 ≈ 30, which clearly exceeds the recommended minimum. Third, the internal consistency of each scale was calculated, as well as the correlation of each item with the total score, corrected to assess the internal discrimination of the test. Fourth, factorial invariance by gender was examined. Fifth, to evaluate the convergent validity of the Spanish version of the CSAS, correlations with the TMMS-24, CHASO, and EHC-PS were analyzed under a hierarchical theoretical framework linking attitude with competence. It was proposed that attitudes toward communication do not operate in isolation, but are supported by emotional maturity (measured by the TMMS-24), manifested in general social interactions (measured by the CHASO), and consolidated in specific clinical communication competencies (measured by the EHC-PS). Thus, significant and positive correlations were hypothesized between the PAS subscale and the scores of the three instruments, as well as inverse (negative) correlations with the NAS subscale, demonstrating that a favorable attitude toward learning these skills converges with more interpersonally competent psychological and behavioral profiles.

Sixth, Pearson correlations were obtained between the CSAS and its dimensions with the two additional scales and their respective dimensions to evaluate the evidence of convergent and discriminant validity. Additionally, the correlation between age and the CSAS was explored. Seventh, evidence of differential validity in the CSAS by gender was investigated using a Student’s t-test. Lastly, test norms were calculated, including percentile scores, Z scores, normalized Z scores, and T scores.

All analyses were conducted using SPSS V. 23 and the AMOS V. 23 program (56).

## 3 Results

### 3.1 Descriptive analyses

Table 2 presents the descriptive statistics for the measures evaluated in the study. For all measures, skewness did not exceed | ± 2|, and kurtosis remained below | ± 7| (57). Additionally, internal consistency (Cronbach’s *α*) was reported for the measures of Emotional Intelligence (TMMS), Social Skills (CHASO), and Communication (EHC-PS). Overall, the values were deemed acceptable, as they exceeded the threshold of 0.70 (53), except for the “Coping with Ridicule” subscale (CHASO) and the “Social Skills” subscale (EHC-PS). Therefore, caution should be exercised when interpreting the results for these two subscales.

### 3.2 Internal validity evidence of the scale

**Figure 1:**
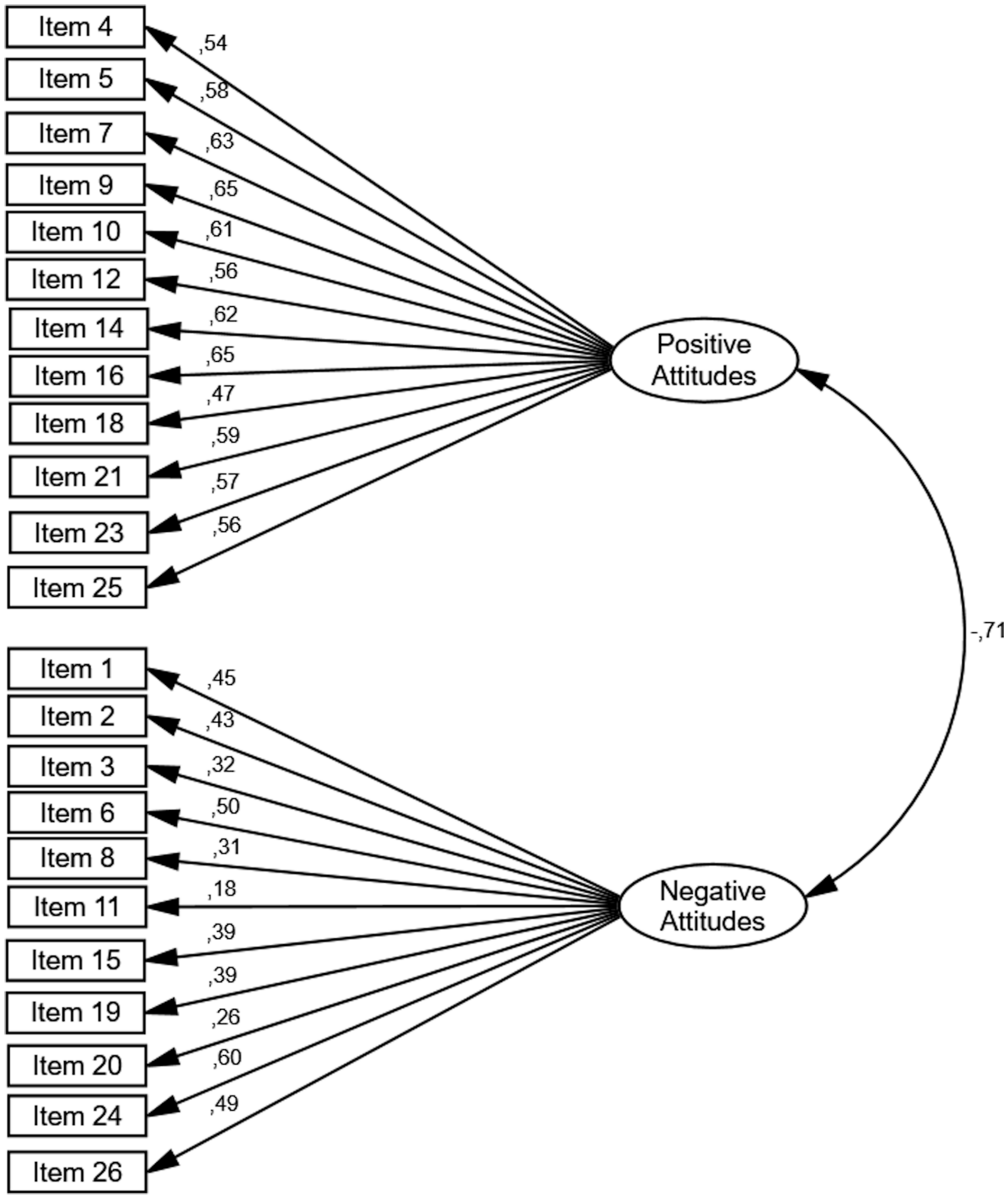
Two-factor model for the CSAS.

A confirmatory model with two correlated factors, encompassing all 26 items, was estimated (see Figure 1). The model did not meet multivariate normality based on the Bollen-Stine bootstrap test (58) (*p* = 0.005), prompting the use of unweighted least squares estimation, which does not require normality. Table 3 presents the goodness-of-fit indices for the estimated model, showing a moderate fit to the data. Items with factor loadings below the recommended minimum of 0.40 (53) and whose removal increased the reliability of the two factors were eliminated. Specifically, three items were removed, and the two-factor model was re-estimated without them (53). Once again, multivariate normality was not met, and the model was estimated using unweighted least squares. As shown in Figure 1, most factor loadings exceed 0.40, and the correlation between the two factors is high and negative (−0.71), which is consistent with the expectation that the two factors measure positive and negative attitudes. The final Spanish version of the administered questionnaire can be consulted in Supplementary Material.

**Table 3:** Goodness-of-fit indices for the models.

Model	*χ*^2^*/*df	GFI	NFI	PGFI	PNFI	SRMR
CSAS 26 items	2.39	0.952	0.875	0.809	0.803	0.061
CSAS 23 items	1.82	0.967	0.920	0.802	0.832	0.059

*χ*^2^/df, chi square; GFI, Goodness-of-Fit Index; NFI, Normed Fit Index; PGFI, Parsimony Goodness-of-Fit Index; PNFI, Parsimony Normed Fit Index; SRMR, Standardized Root Mean Square Residual.

### 3.3 Reliability evidence of the scale

Internal consistency was calculated for the two CSAS subscales using Cronbach’s *α*. As shown in Table 2, the internal consistency for the PAS subscale is acceptable (0.852), while the NAS subscale falls short of the 0.70 minimum threshold (0.632). We also calculated McDonald’s *ω* reliability index, obtaining slightly higher values: 0.863 for the PAS and 0.667 for the NAS. The internal discrimination of each subscale was analyzed by computing the corrected item-total correlation for each item within its respective subscale. In all cases, the internal discrimination values were > 0.20, suggesting that the items demonstrate adequate internal discrimination (53).

### 3.4 Evidence of factorial invariance by gender

**Figure 2:**
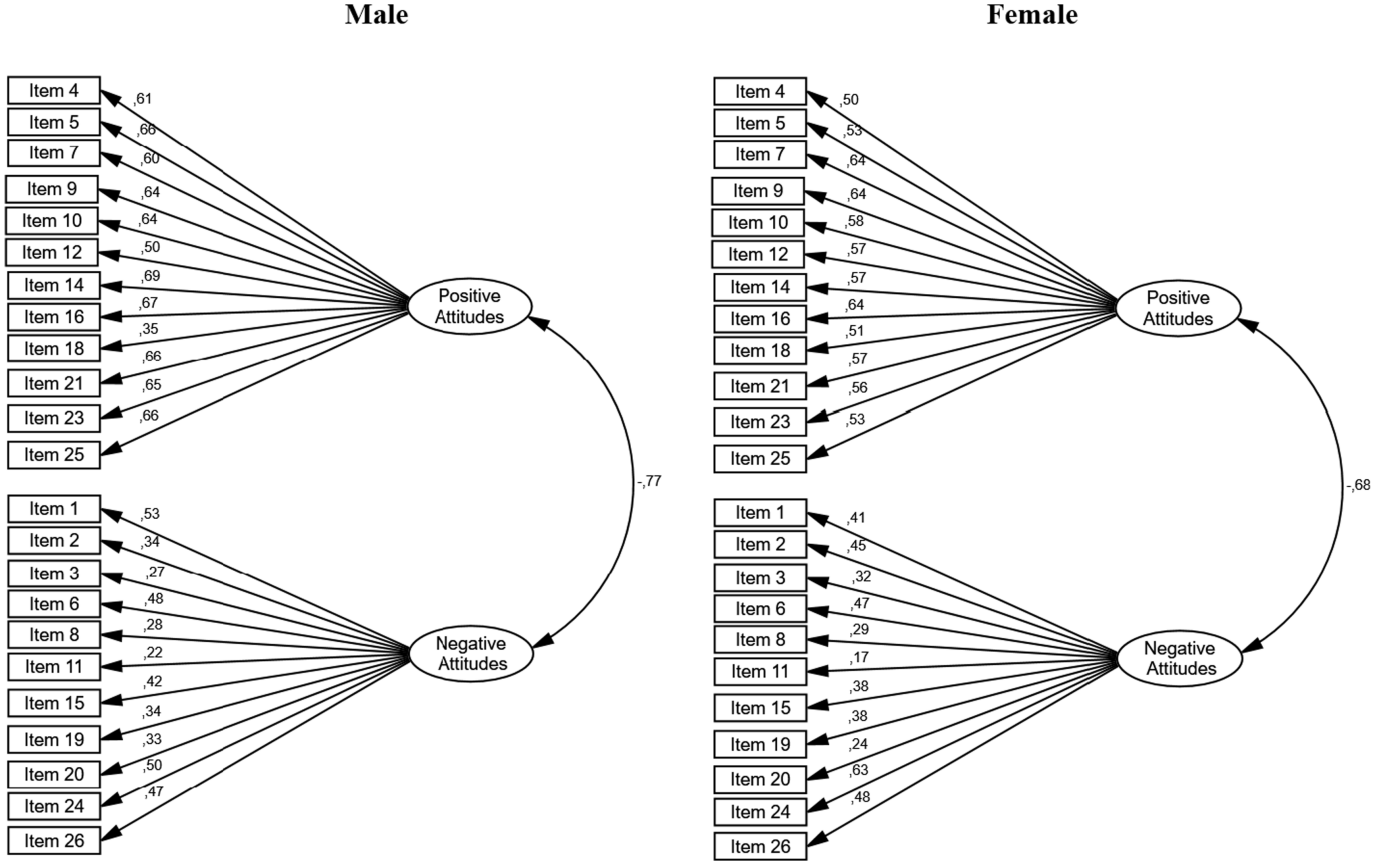
Two-factor model for CSAS by gender.

The analysis followed the approach proposed by Vandenberg and Lance (59), comparing nested models to determine whether they are equivalent across independent groups. The models tested were as follows: (1) an unconstrained model, where factor loadings, variance–covariance matrices, and error variances are allowed to differ; (2) metric invariance, where the factor loadings are constrained to be equal across groups; (3) strong invariance, where the variance–covariance matrices are constrained to equality; and (4) strict invariance, where error variances are also constrained to equality. Neither of the models for men or women exhibited multivariate normality, as indicated by the Bollen-Stine bootstrap (58) (*p* = 0.005), so they were estimated using unweighted least squares. Table 4 presents the goodness-of-fit indices, and Figure 2 displays the comparison of both models. The Δ*χ*^2^ increase was statistically significant (*p* < 0.001) and ΔSRMR > 0.01 (60) for all comparisons, suggesting that the factorial structure differs between genders. The factor loadings and correlations between the two factors vary, with males exhibiting a stronger relationship between factors (−0.77) compared to females (−0.68).

**Table 4:** Factorial invariance for sex in the CSAS.

Specified model	*χ*^2^	df	*χ*^2^/df	GFI	NFI	SRMR
Model A. Unconstrained	530.81	94	5.65	0.957	0.894	0.074
Model B. equal factor loadings (structural weights)	627.40	73	8.59	0.949	0.875	0.090
Model C. Equal factor loadings and variance–covariance matrix (structural covariances)	699.83	70	10.00	0.943	0.861	0.107
Model D. Equal factor loadings and variance–covariance matrix, and error variance (measurement residuals)	784.46	47	10.69	0.936	0.844	0.087

Model comparison	Δ*χ*^2^	Δ*df*	*p*			ΔSRMR
Models A and B (metric invariance)	96.59	21	< 0.001			0.016
Models B and C (strong metric invariance)	72.43	3	< 0.001			0.017
Models C and D (strict metric invariance)	84.6	23	< 0.001			0.020

### 3.5 Evidence of convergent and discriminant validity

Pearson correlations were computed between the two subscales of the Communication Skills Attitude Scale (CSAS) and the Trait Meta-Mood Scale (TMMS), the Social Skills Questionnaire (CHASO), and the Communication Skills in Health Professionals Scale (EHC-PS). The results are summarized in Table 5. A minimum correlation threshold of 0.30 was established, as this is considered a medium effect size for correlation according to Cohen (61). The *post hoc* statistical power (1 – *β*) was calculated for a sample of *N* = 792 with a significance level (*α*) of 0.05 and an effect size of 0.30. A power of 1.00 was obtained, which is clearly superior to the recommended minimum of 0.90 (53).

**Table 5:** Pearson correlations (*R*) y *R^2^* among CSAS, Age, TMMS, CHASO, and EHC-PS.

Measure	Positive attitudes		Negative attitudes	
	*R*	*R^2^*	*R*	*R^2^*
Age	0.04	0.00	−0.08	0.01
Emotional attention (TMMS)	0.23	0.05	−0.15	0.02
Emotional clarity (TMMS) (TMMS)	0.21	0.04	−0.18	0.03
Emotional repair (TMMS)	0.27	0.07	−0.15	0.02
Emotional intelligence (TMMS)	**0.34**	0.12	−0.23	0.05
Interacting with strangers (CHASO)	0.22	0.05	−0.12	0.01
Positive feelings (CHASO)	**0.32**	0.10	−0.21	0.04
Coping with criticism (CHASO)	0.23	0.05	−0.19	0.04
Interacting with acquaintances (CHASO)	0.08	0.01	0.01	0.00
Calmness in the face of criticism (CHASO)	0.24	0.06	−0.14	0.02
Public SPEAKING/INTERACTING WITH SUPERIORS (CHASO)	0.22	0.05	−0.15	0.02
Coping with ridicule (CHASO)	0.14	0.02	0.01	0.00
Defending rights (CHASO)	0.22	0.05	−0.09	0.01
Apologizing (CHASO)	**0.38**	0.14	**−0.33**	0.11
Refusing requests (CHASO)	0.19	0.04	−0.09	0.01
Total social skills (CHASO)	**0.34**	0.12	−0.19	0.04
Empathy (EHC-PS)	**0.51**	0.26	**−0.35**	0.12
Informative communication (EHC-PS)	**0.46**	0.21	**−0.41**	0.17
Respect (EHC-PS)	**0.43**	0.18	**−0.32**	0.10
Social Skill (EHC-PS)	0.26	0.07	−0.18	0.03
Total communication skills (EHC-PS)	**0.52**	0.27	**−0.40**	0.16

Correlations ≥ | ± 0.07| are statistically significant at the 5% level. Correlations ≥ 0.30 are in bold.

Regarding convergent validity, highlighted correlations in Table 5 indicate that the Positive Attitude Subscale (PAS) exhibits significant positive correlations with measures of Emotional Intelligence (TMMS), Positive Feelings (CHASO), Apologizing (CHASO), Social Skills (CHASO), Empathy (EHC-PS), Informative Communication (EHC-PS), Respect (EHC-PS), and Communication Skills (EHC-PS). Conversely, the Negative Attitude Subscale (NAS) shows significant negative correlations with Apologizing (CHASO), Empathy (EHC-PS), Informative Communication (EHC-PS), Respect (EHC-PS), and Communication Skills (EHC-PS). The *R^2^* value is also provided to observe the variance shared by both variables; as can be seen, the higher the correlation between the means, the greater the shared variance. These findings clearly indicate that the CSAS demonstrates adequate convergent validity when compared to similar measures in other scales.

#### 3.5.1 Relationship with age

Pearson correlations were computed between the age of the participants and the two subscales of the Communication Skills Attitude Scale (CSAS), yielding a null correlation (Table 5). This indicates that the communication skills attitudes measured by the CSAS are independent of the participants’ age.

### 3.6 Evidence of differential validity

Although the analysis indicated an absence of strict factorial invariance across genders, this does not preclude the examination of mean differences. While factorial invariance assesses the structural configuration, a Student’s t-test evaluates whether the scores of the latent factors exhibit equivalent means, making them complementary analyses. Mean differences were calculated using a Student’s t-test for the two subscales of the Communication Skills Attitude Scale (CSAS). The results are presented in Table 6. Statistically significant differences were found for both subscales, with males scoring higher. The effect size was also calculated using Cohen’s *d* (60), where values of 0.20 are considered a small effect, 0.50 a medium effect, and 0.80 a large effect. In our study, the mean differences between sexes exhibited medium effect sizes (ranging from 0.34 to 0.49), indicating clear differences in communication skills attitudes between genders, with higher scores for males.

**Table 6:** Descriptive statistics, mean differences, and effect size for the CSAS.

Measure	Male M (SD)	Female M (SD)	*t*-test	Cohen’s *d*
Positive attitudes	51.17 (6.46)	53.31 (5.51)	*t*_790_ = −4.34, *p* < 0.001	−0.34
Negative attitudes	22.70 (5.36)	21.24 (4.94)	*t*_790_ = 5.67, *p* < 0.001	0.49
Item 4 (PAS)	4.28 (0.94)	4.54 (0.67)	*t*_790_ = 3.42, *p* = 0.001	0.29
Item 5 (PAS)	4.60 (0.75)	4.75 (0.55)	*t*_790_ = 2.48, *p* = 0.014	0.21
Item 7 (PAS)	4.45 (0.75)	4.58 (0.64)	*t*_790_ = 2.09, *p* = 0.038	0.18
Item 9 (PAS)	4.45 (0.77)	4.64 (0.61)	*t*_790_ = 3.03, *p* = 0.003	0.26
Item 10 (PAS)	4.41 (0.71)	4.59 (0.65)	*t*_790_ = 2.95, *p* = 0.003	0.26
Item 12 (PAS)	3.76 (0.93)	3.94 (0.95)	*t*_790_ = 2.11, *p* = 0.034	0.18
Item 14 (PAS)	4.36 (0.86)	4.57 (0.68)	*t*_790_ = 3.02, *p* = 0.003	0.26
Item 16 (PAS)	4.23 (0.91)	4.48 (0.73)	*t*_790_ = 3.37, *p* = 0.001	0.29
Item 18 (PAS)	3.52 (1.06)	3.75 (1.16)	*t*_790_ = 2.37, *p* = 0.018	0.20
Item 21 (PAS)	4.48 (0.75)	4.58 (0.73)	*t*_790_ = 1.66, *p* = 0.098	0.14
Item 23 (PAS)	4.20 (0.84)	4.33 (0.82)	*t*_790_ = 1.77, *p* = 0.077	0.15
Item 25 (PAS)	4.44 (0.73)	4.56 (0.72)	*t*_790_ = 1.96, *p* = 0.051	0.17
Item 1 (NAS)	1.20 (0.46)	1.14 (0.40)	*t*_790_ = 1.62, *p* = 0.107	0.14
Item 2 (NAS)	1.39 (0.80)	1.26 (0.62)	*t*_790_ = 1.94, *p* = 0.053	0.17
Item 3 (NAS)	2.80 (1.22)	2.60 (1.13)	*t*_790_ = 2.01, *p* = 0.044	0.17
Item 6 (NAS)	2.09 (1.10)	1.61 (0.94)	*t*_790_ = 5.63, *p* < 0.001	0.48
Item 8 (NAS)	2.73 (1.33)	2.32 (1.34)	*t*_790_ = 3.65, *p* < 0.001	0.31
Item 11 (NAS)	2.91 (1.10)	2.88 (1.11)	*t*_790_ = 0.34, *p* = 0738	0.03
Item 15 (NAS)	2.33 (1.05)	2.15 (1.06)	*t*_790_ = 1.96, *p* = 0.051	0.17
Item 19 (NAS)	1.86 (1.14)	1.47 (0.89)	*t*_790_ = 4.06, *p* < 0.001	0.35
Item 20 (NAS)	2.29 (1.11)	2.29 (1.09)	*t*_790_ = 0.03, *p* = 0.980	0.00
Item 24 (NAS)	2.30 (1.05)	2.04 (1.05)	*t*_790_ = 2.91, *p* = 0.004	0.25
Item 26 (NAS)	1.80 (1.12)	1.48 (0.87)	*t*_790_ = 3.49, *p* = 0.001	0.30

Positive Attitude Subscale (PAS), Negative Attitude Subscale (NAS).

Mean differences were also calculated for each of the items comprising the scale. As can be seen in Table 6, only one item has a medium effect size (0.48), specifically item 6. Some items (items 4, 8, 16, 19, and 26) show effect sizes close to 0.30. This result clearly indicates that the scale does not function identically across both sexes.

### 3.7 Norms

Table 7 presents the normative scores for the assessed participants. For each raw score, the percentile rank, standardized score (Z), normalized Z score, and T score (M = 50, SD = 10) were calculated. These data will enable future evaluators to determine the relative position of their subjects compared to a primarily health sciences university sample. Norms were computed separately for each gender for both subscales of the CSAS.

**Table 7:** Norms for the CSAS for Positive Attitudes (PAS) and Negative Attitudes (NAS).

Percentile	Z_n_	*T*	PAS male	Z PAS male	NAS male	Z NAS female	PAS female	Z PAS female	NAS female	Z NAS female
1	−2.33	26.70	36.74	−2.23	11.74	−2.23	38.20	−2.72	12.00	−1.87
5	−1.64	33.60	39.00	−1.88	15.70	−1.49	43.00	−1.85	14.00	−1.47
10	−1.28	37.20	41.00	−1.57	17.00	−1.25	45.00	−1.49	15.00	−1.26
15	−1.04	39.60	43.00	−1.26	18.00	−1.06	47.00	−1.13	16.00	−1.06
20	−0.84	41.60	45.00	−0.96	19.00	−0.88	48.00	−0.95	17.00	−0.86
25	−0.67	43.30	47.00	−0.65	20.00	−0.69	49.00	−0.76	17.00	−0.86
30	−0.52	44.80	48.00	−0.49	20.00	−0.69	51.00	−0.40	18.00	−0.66
35	−0.39	46.10	49.00	−0.34	21.00	−0.50	52.00	−0.22	19.00	−0.45
40	−0.25	47.50	50.00	−0.18	21.60	−0.39	53.00	−0.04	19.00	−0.45
45	−0.13	48.70	51.00	−0.03	22.30	−0.26	54.00	0.14	20.00	−0.25
50	0.00	50.00	52.00	0.13	23.00	−0.13	55.00	0.32	21.00	−0.05
55	0.13	51.30	53.00	0.28	24.00	0.06	55.00	0.32	22.00	0.15
60	0.25	52.50	54.00	0.44	25.00	0.24	56.00	0.51	22.00	0.15
65	0.39	53.90	55.00	0.59	26.00	0.43	57.00	0.69	23.00	0.36
70	0.52	55.20	56.00	0.75	27.00	0.62	57.00	0.69	24.00	0.56
75	0.67	56.70	57.00	0.90	27.00	0.62	58.00	0.87	25.00	0.76
80	0.84	58.40	58.00	1.06	29.00	0.99	58.00	0.87	26.00	0.96
85	1.04	60.40	58.00	1.06	30.00	1.18	59.00	1.05	27.00	1.17
90	1.28	62.80	59.00	1.21	31.00	1.36	60.00	1.23	28.00	1.37
95	1.64	66.40	60.00	1.37	33.00	1.74	60.00	1.23	30.00	1.77
99	2.33	73.30	60.00	1.37	35.26	2.16	60.00	1.23	30.00	1.77
N			173	173	173	173	619	619	619	619
Mean	0.00	50.00	51.17	0.00	23.70	0.00	53.31	0.02	21.24	0.00
SD	1.00	10.00	6.46	1.00	5.36	1.00	5.51	1.00	4.94	1.00

## 4 Discussion

This study presents the validation of the CSAS within a sample of 792 university students enrolled in health science programs. Following the elimination of three inadequate items, the internal structure of the scale demonstrated a satisfactory fit (GFI = 0.967, SRMR = 0.059) to a model comprising two negatively correlated factors (−0.71): one representing positive attitudes (PAS) and the other representing negative attitudes (NAS). Consistent with these results, several authors have reported a negative relationship between these two factors (26, 62). Conversely, other researchers propose a three-factor structure that includes learning, importance, and respect (63).

In terms of the reliability of our validation, the Positive Attitudes subscale (PAS) exhibits satisfactory reliability (*α* = 0.852, *ω* = 0.863), while the Negative Attitudes subscale (NAS) demonstrates lower reliability (α = 0.632, ω = 0.667). Nonetheless, both subscales reflect adequate internal discrimination. In the original study, both subscales reported values exceeding 0.80 (20). However, prior research has identified consistency values comparable to those observed in our study for the NAS subscale (Cronbach’s α ranging from 0.61 to 0.68) (28, 29, 64, 65), suggesting that the NAS subscale generally exhibits less consistency than the PAS. This fact should be kept in mind when interpreting the total NAS score, as it is inherently less reliable than the other subscale. Consequently, the total NAS score values should be interpreted with caution, taking into account a certain margin of error in the assessment and its future implications.

Additionally, our analysis revealed significant invariance between the two factors based on sex, as evidenced by differing factor loadings and correlations between the Positive Attitudes subscale (PAS) and the Negative Attitudes subscale (NAS) across genders. Notably, the correlation between PAS and NAS is stronger for males (−0.77) compared to females (−0.68). This finding is particularly noteworthy, as there are no extant studies that have undertaken a similar examination of the scale, thereby constituting a novel contribution to the existing body of literature.

Regarding the evidence of convergent validity, both subscales exhibit adequate convergence with analogous measures, specifically TMMS, CHASO, and EHC-PS. Previous research has not explored this aspect of validity, thus rendering this finding a significant contribution of our study.

Moreover, we identified evidence of differential validity, as male participants scored higher on both subscales compared to their female counterparts. However, no correlation was observed between age and the CSAS measures. While some studies report no significant differences based on sex in this scale (57), others suggest that women demonstrate more favourable attitudes toward learning in health sciences, which contradicts our results (21, 22, 66–68).

Lastly, we provide normative data for the two subscales of the CSAS, differentiated by sex, to assist future evaluators in their assessments. Notably, prior adaptations of this scale did not furnish such normative data. A potential factor explaining these sex differences could be the variation in social desirability bias between genders. Future lines of research should investigate which sex exhibits a higher propensity for social desirability on the CSAS.

### 4.1 Limitations

The primary limitation of the current study resides in the characteristics of the evaluated sample, which is exclusively comprised of undergraduate students in Health Sciences. Future research should endeavour to assess individuals from a broader range of academic disciplines. Furthermore, the sample is predominantly composed of younger individuals, highlighting the need for investigations into the scale’s performance across various age groups. Additionally, the sample exhibits a higher proportion of females compared to males, which may introduce potential bias into the results. It would also be beneficial to examine changes in scale scores following interventions aimed at enhancing social and communication skills, thus providing empirical evidence of the scale’s sensitivity to change. The sample selection method was non-random and incidental, which limits the generalizability of the obtained results. In the future, it would be highly beneficial to conduct studies aiming to replicate these findings using representative samples in order to mitigate this bias.

## 5 Conclusion

The CSAS exhibits strong psychometric properties, making it suitable for assessing social and communication skills, both at a general level and specifically within the domain of Health Sciences. This study contributes significantly to the existing body of literature by exploring differential and convergent validity, as well as factorial invariance across gender. Moreover, we present normative data for the scale, which could serve as a valuable resource for its application in practical clinical and educational contexts.

## Data Availability

The original contributions presented in the study are included in the article/supplementary material, further inquiries can be directed to the corresponding author.

## References

[1] Mercan N. Examination of nursing students’ communication with life examples: a mixed method study. J Educ Res Nurs. (2023) 20:232–40. doi: 10.14744/jern.2021.21291

[2] Busquets-Guiu L Gomar-Sancho C Paredes-Zapata D. Formación en habilidades de comunicación en estudiantes de medicina de la Facultad de Medicina y Ciencias de la Salud de la Universitat de Barcelona. Rev Fund Educ Med. (2020) 23:97. doi: 10.33588/fem.232.1051

[3] Guidi C Traversa C. Empathy in patient care: from ‘clinical empathy’ to ‘empathic concern’. Med Health Care Philos. (2021) 24:573–85. doi: 10.1007/s11019-021-10033-4, 34196934 PMC8557158

[4] Moudatsou M Stavropoulou A Philalithis A Koukouli S. The role of empathy in health and social care professionals. Healthcare. (2020) 8:26. doi: 10.3390/healthcare8010026, 32019104 PMC7151200

[5] Foster K Fethney J Kozlowski D Fois R Reza F McCloughen A. Emotional intelligence and perceived stress of Australian pre-registration healthcare students: a multi-disciplinary cross-sectional study. Nurse Educ Today. (2018) 66:51–6. doi: 10.1016/j.nedt.2018.04.001, 29665505

[6] McCloughen A Foster K. Nursing and pharmacy students’ use of emotionally intelligent behaviours to manage challenging interpersonal situations with staff during clinical placement: a qualitative study. J Clin Nurs. (2017) 27:2699–709. doi: 10.1111/jocn.13865, 28426909

[7] Bas-Sarmiento P Fernández-Gutiérrez M Baena-Baños M Romero-Sánchez JM. Efficacy of empathy training in nursing students: a quasi-experimental study. Nurse Educ Today. (2017) 59:59–65. doi: 10.1016/j.nedt.2017.08.012, 28945994

[8] Håkansson Eklund J Holmström IK Ollén Lindqvist A Sundler AJ Hochwälder J Marmstål Hammar L. Empathy levels among nursing students: a comparative cross-sectional study. Nurs Open. (2019) 6:983–9. doi: 10.1002/nop2.280, 31367422 PMC6650686

[9] Ferrández-Antón T Ferreira-Padilla G del Pino Casado R Ferrández-Antón P Baleriola-Júlvez J Martínez-Riera JR. Communication skills training in undergraduate nursing programs in Spain. Nurse Educ Pract. (2020) 42:102653. doi: 10.1016/j.nepr.2019.102653 31734517

[10] Brouwers M Rasenberg E van Weel C Laan R van Weel-Baumgarten E. Assessing patient-centred communication in teaching: a systematic review of instruments. Med Educ. (2017) 51:1103–17. doi: 10.1111/medu.13375, 28762538 PMC5655924

[11] Langberg EM Dyhr L Davidsen AS. Development of the concept of patient-centredness – a systematic review. Patient Educ Couns. (2019) 102:1228–36. doi: 10.1016/j.pec.2019.02.023, 30846206

[12] Sturgiss EA Peart A Richard L Ball L Hunik L Chai TL . Who is at the Centre of what? A scoping review of the conceptualisation of ‘centredness’ in healthcare. BMJ Open. (2022) 12:e059400. doi: 10.1136/bmjopen-2021-059400, 35501096 PMC9062794

[13] Escribano S Juliá-Sanchis R García-Sanjuán S Congost-Maestre N Cabañero-Martínez MJ. Psychometric properties of the attitudes towards medical communication scale in nursing students. PeerJ. (2021) 9:e11034. doi: 10.7717/peerj.11034, 34113481 PMC8162233

[14] Axboe MK Christensen KS Kofoed PE Ammentorp J. Development and validation of a self-efficacy questionnaire (SE-12) measuring the clinical communication skills of health care professionals. BMC Med Educ. (2016) 16:272. doi: 10.1186/s12909-016-0798-7, 27756291 PMC5069791

[15] Mora-Pelegrín M Montes-Berges B Aranda M Vázquez MA Armenteros-Martínez E. The empathic capacity and the ability to regulate it: construction and validation of the empathy management scale (EMS). Healthcare. (2021) 9:587. doi: 10.3390/healthcare9050587, 34063535 PMC8156607

[16] Han S Yoo J Kang K. Development and validation of the therapeutic communication scale in nursing students. Healthcare. (2024) 12:394. doi: 10.3390/healthcare12030394, 38338279 PMC10855793

[17] Soares SF Carvalho Moura EC Lopez V Peres AM. Professional nursing communication competence: theoretical procedures for instrument development and pilot test. J Nurs Manag. (2021) 29:1496–507. doi: 10.1111/jonm.13283, 33548089

[18] Leal C Tirado S Rodríguez-Marín J van-der Hofstadt CJ. Creación de la escala sobre habilidades de comunicación en profesionales de la salud, EHC-PS. An Psicol. (2015) 32:49. doi: 10.6018/analesps.32.1.184701

[19] Leal-Costa C Tirado-González S Rodríguez-Marín J van-der- Hofstadt-Román CJ. Psychometric properties of the health professionals communication skills scale (HP-CSS). Int J Clin Health Psychol. (2016) 16:76–86. doi: 10.1016/j.ijchp.2015.04.001 30487852 PMC6225029

[20] Rees C Sheard C Davies S. The development of a scale to measure medical students’ attitudes towards communication skills learning: the communication skills attitude scale (CSAS). Med Educ. (2002) 36:141–7. doi: 10.1046/j.1365-2923.2002.01072.x, 11869441

[21] Molinuevo B Torrubia R. Validation of the Catalan version of the communication skills attitude scale (CSAS) in a cohort of south European medical and nursing students. Educ Health. (2011) 24:499. doi: 10.4103/1357-6283.101458 21710418

[22] Rees C Sheard C. The relationship between medical students’ attitudes towards communication skills learning and their demographic and education-related characteristics. Med Educ. (2002) 36:1017–27. doi: 10.1046/j.1365-2923.2002.01333.x, 12406261

[23] Ahn S Yi YH Ahn DS. Developing a Korean communication skills attitude scale: comparing attitudes between Korea and the West. Med Educ. (2009) 43:246–53. doi: 10.1111/j.1365-2923.2008.03271.x, 19250351

[24] Laurence B Bertera EM Feimster T Hollander R Stroman C. Adaptation of the communication skills attitude scale (CSAS) to dental students. J Dent Educ. (2012) 76:1629–38. doi: 10.1002/j.0022-0337.2012.76.12.tb05426.x, 23225682

[25] Przymuszała P Cerbin-Koczorowska M Marciniak-Stępak P Zielińska-Tomczak Ł Piszczek M Jasiński J . Affective and cognitive components of students’ attitudes towards communication learning - validation of the communication skills attitude scale in a cohort of polish medical students. BMC Med Educ. (2021) 21:190. doi: 10.1186/s12909-021-02626-7, 33794870 PMC8017827

[26] Busch AK Rockenbauch K Schmutzer G Brähler E. Do medical students like communication? Validation of the German CSAS (communication skills attitude scale). GMS Z Med Ausbild. (2015) 32:Doc11. doi: 10.3205/zma000953 25699103 PMC4330630

[27] Ferrari A Terzoni S Ferrara P Provenzano M Destrebecq A. L’attitudine degli studenti di infermieristica a sviluppare adeguate abilità comunicative: validazione in italiano della communication skills attitude scale (CSAS). L’Infermiere. (2017) 54:e7–e15.

[28] Harlak H Dereboy Ç Gemalmaz A. Validation of a Turkish translation of the communication skills attitude scale with Turkish medical students. Educ Health. (2008) 21:55 19034831

[29] Mohamad-Isa MZ Mohamed-Yassin MS Badlishah-Sham SF Baharudin N Ramli AS. Cross-cultural adaptation and psychometric properties of the Malay version of the communication skills attitude scale (CSAS) among medical students in Malaysia. Int J Environ Res Public Health. (2021) 18:3778. doi: 10.3390/ijerph18073778, 33916335 PMC8038565

[30] Panczyk M Iwanow L Zarzeka A Jaworski M Gotlib J. Communication skills attitude scale: a translation and validation study in a sample of registered nurses in Poland. BMJ Open. (2019) 9:e028691. doi: 10.1136/bmjopen-2018-028691, 31072864 PMC6527998

[31] Santos JLG Copelli FHS Balsanelli AP Sarat CNF Menegaz JC Trotte LAC . Interpersonal communication competence among nursing students. Rev Lat Am Enfermagem. (2019) 27:e3207. doi: 10.1590/1518-8345.3226.3207, 31664414 PMC6818656

[32] Gutiérrez-Puertas L Márquez Hernández VV Gutiérrez-Puertas V Granados-Gámez G Aguilera-Manrique G. Interpersonal communication, empathy, and stress perceived by nursing students who use social networks. J Adv Nurs. (2020) 76:2610–7. doi: 10.1111/jan.14494, 32803905

[33] Ezeala MIO Volk J. Relationships between undergraduate medical students’ attitudes toward communication skills learning and demographics in Zambia: a survey-based descriptive study. J Educ Eval Health Prof. (2023) 20:16. doi: 10.3352/jeehp.2023.20.16, 37385684 PMC10315251

[34] Balcar J Dokoupilová L. Communication and language skills pay off, but not everybody needs them. Int J Sociol Lang. (2021) 2021:59–93. doi: 10.1515/ijsl-2020-0021

[35] Liu J Yuan K Lin X Zhu W. What learning strategies influence higher-order learning behaviours of medical students? Ann Med. (2023) 55:1. doi: 10.1080/07853890.2023.2205166, 37171217 PMC10184605

[36] Wright KB Bylund C Ware J Parker P Query JL Baile W. Medical student attitudes toward communication skills training and knowledge of appropriate provider-patient communication: a comparison of first-year and fourth-year medical students. Med Educ Online. (2006) 11:4594. doi: 10.3402/meo.v11i.4594, 28253797

[37] Datta M. Gender diverse attitudes fade with time: a longitudinal study on communication skills and professionalism among MBBS students in the era of AETCOM training. J Multidiscip Res Healthc. (2026) 12:39–49. doi: 10.15415/jmrh.2025.121005

[38] Brotons P Virumbrales M Elorduy M Díaz de Castellví S Mezquita P Gené E . Improvement of medical students' performance in simulated patient interviews by pre-clinical communication training. Int J Med Educ. (2022) 13:148–53. doi: 10.5116/ijme.6299.c15f, 35716402 PMC9902174

[39] Harlak H Dereboy Ç Gemalmaz A. Validation of the communication skills attitude scale (CSAS) in a Turkish medical student sample: the problem of social desirability. Med Teach. (2008) 30:e164–8. doi: 10.1080/01421590802144274

[40] Bugaj TJ Cranz A Junne F Erschens R Herzog W Nikendei C. Communication skills training in medical education: the effect of subject-specific characteristics and gender on students' attitude. GMS J Med Educ. (2017) 34:1095. doi: 10.3205/zma001095

[41] Claramita M Utarini A Soebono H Van Dalen J Van der Vleuten CP. Doctor-patient communication in a southeast Asian setting: the conflict between traditional and modern expectations. Med Educ. (2011) 45:298–307. doi: 10.1111/j.1365-2923.2010.03874.x, 20658353 PMC3074074

[42] Reith-Hall E Montgomery P. Communication skills training for improving the communicative abilities of student social workers: a systematic review. Campbell Syst Rev. (2023) 19:e1309. doi: 10.1002/cl2.1309, 36911865 PMC9949884

[43] Brundiers K Wiek A. Beyond interpersonal competence: teaching and learning professional skills in sustainability. Educ Sci. (2017) 7:39. doi: 10.3390/educsci7010039

[44] Salavera C Usán P. Exploración de la dimensionalidad y las propiedades psicométricas de la escala EIS de inteligencia emocional. Rev CES Psicol. (2019) 12:50–66. doi: 10.21615/cesp.12.3.4

[45] Caballo V Salazar I. Development and validation of a new social skills assessment instrument: the social skills questionnaire (CHASO). Behav Psychol. (2017) 25:5–24.

[46] Cegala DJ Coleman MT Turner JW. The development and partial assessment of the medical communication competence scale. Health Commun. (1998) 10:261–88. doi: 10.1207/s15327027hc1003_5, 16370986

[47] Salovey P Mayer J. Emotional intelligence. Imagin Cogn Pers. (1990) 9:185–211. doi: 10.2190/DUGG-P24E-52WK-6CDG

[48] Leal-Costa C Tirado González S Ramos-Morcillo AJ Ruzafa-Martínez M Díaz Agea JL van-der Hofstadt Román CJ. Communication skills and professional practice: does it increase self-efficacy in nurses? Front Psychol. (2020) 11:1169. doi: 10.3389/fpsyg.2020.01169, 32595561 PMC7304242

[49] Brislin RW. Back-translation for cross-cultural research. J Cross-Cult Psychol. (1970) 1:185–216. doi: 10.1177/135910457000100301

[50] Bentler PM Bonett DG. Significance tests and goodness of fit in the analysis of covariance structures. Psychol Bull. (1980) 88:588–606. doi: 10.1037/0033-2909.88.3.588

[51] Joreskog KG Sorbom D. LISREL 8: User’s Guide. Chicago, IL: Scientific Software International (1993).

[52] Hu L Bentler PM. Cutoff criteria for fit indexes in covariance structure analysis: conventional criteria versus new alternatives. Struct Equ Model. (1999) 6:1–55. doi: 10.1080/10705519909540118

[53] Hair JF Anderson RE Tatham RL Black WC. Análisis Multivariante. 5th ed. Madrid: Prentice Hall (1999).

[54] James LR Mulaik SA Brett JM. Causal Analysis: Models, Assumptions and Data. 2nd ed. Thousand Oaks, CA: SAGE Publications (1982).

[55] Byrne BM. Structural Equation Modeling with AMOS: Basic Concepts, Applications, and Programming. Mahwah, NJ: Lawrence Erlbaum Associates (2001).

[56] Arbuckle JL. Amos 23.0 User’s Guide. Crawfordville, FL: Amos Development Corporation (2014).

[57] West SG Finch JF Curran PJ Hoyle RH. "Structural equation models with nonnormal variables: problems and remedies". In: Hoyle R, editor. Structural Equation Modeling: Concepts, Issues, and Applications. Thousand Oaks, CA: Sage (1995). p. 56–75.

[58] Bollen K Long JS, editors. Testing Structural Equation Models. Newbury Park, CA: SAGE Publications (1993). p. 111–35.

[59] Vandenberg RJ Lance CE. A review and synthesis of the measurement invariance literature: suggestions, practices, and recommendations for organization research. Organ Res Methods. (2000) 3:4–70. doi: 10.1177/109442810031002

[60] Cheung GW Rensvold RB. Evaluating goodness-of-fit indexes for testing measurement invariance. Struct Equ Model Multidiscip J. (2002) 9:233–55. doi: 10.1207/s15328007sem0902_5

[61] Cohen J. A power primer. Psychol Bull. (1992) 112:155–9. doi: 10.1037/0033-2909.112.1.155, 19565683

[62] Zhang Y Jiang G Sun Y Zhao X Yu X. Cross-cultural adaptation and psychometric properties of the Chinese version of the communication skills attitude scale among medical students in Liaoning Province, China: a cross-sectional study. BMJ Open. (2018) 8:e020931. doi: 10.1136/bmjopen-2017-020931, 30206076 PMC6144332

[63] Anvik T Gude T Grimstad H Baerheim A Fasmer OB Hjortdahl P. Assessing medical students’ attitudes towards learning communication skills – which components of attitudes do we measure. BMC Med Educ. (2007) 7:4. doi: 10.1186/1472-6920-7-4, 17394673 PMC1851955

[64] Shankar RP Dubey AK Mishra P Deshpande VY Chandrasekhar TS Shivananda PG. Student attitudes towards communication skills training in a medical college in western Nepal. Educ Health. (2006) 19:71–84. doi: 10.1080/13576280500534693, 16531304

[65] Yakhforoshha A Shirazi M Yousefzadeh N Ghanbarnejad A Cheraghi M . Psychometric properties of the communication skills attitude scale (CSAS) measure in a sample of Iranian medical students. J Adv Med Educ Prof. (2018) 6:14–21. doi: 10.30476/JAMP.2018.43343 29344525 PMC5757152

[66] Cleland J Foster K Moffat M. Undergraduate students’ attitudes to communication skills learning differ depending on year of study and gender. Med Teach. (2005) 27:246–51. doi: 10.1080/01421590400029541, 16011948

[67] Lumma-Sellenthin A. Students’ attitudes towards learning communication skills: correlating attitudes, demographic and metacognitive variables. Int J Med Educ. (2012) 3:201–8. doi: 10.5116/ijme.5066.cef9

[68] Wiskin CMD Allan TF Skelton JR. Gender as a variable in the assessment of final year degree-level communication skills. Med Educ. (2004) 38:129–37. doi: 10.1111/j.1365-2923.2004.01746.x, 14871383

