# Supplementary material for: Measuring attitudes toward communication skills in nursing and health sciences: psychometric properties of the CSAS in Spain
Source: Front Public Health. 2026 Jul 13;14:1879823. doi: 10.3389/fpubh.2026.1879823 (PMC13402530; doi:10.3389/fpubh.2026.1879823)
Supplement: Supplementary file 1 [file Supplementary_file_1.docx]

**Supplementary material. CSAS (spanish final version)**

| 1. Para ser un buen enfermero debo tener buenas habilidades comunicativas |
| --- |
| 2. No veo el propósito de aprender habilidades de comunicación |
| 3. Nadie va a suspender la carrera de enfermero por tener malas habilidades comunicativas |
| 4. Desarrollar mi habilidad comunicativa es tan importante como desarrollar mis conocimientos de enfermero |
| 5. Aprender habilidades comunicativas me ha ayudado o me ayudará a respetar a los pacientes |
| 6. No tengo tiempo para aprender habilidades de comunicación |
| 7. Aprender habilidades comunicativas es interesante |
| 8. No me molesto en asistir a las sesiones sobre habilidades de comunicación |
| 9. Aprender habilidades de comunicación ha contribuido o contribuirá a facilitar mi capacidad para trabajar en equipo |
| 10. Aprender habilidades de comunicación ha mejorado mi capacidad para comunicarme con los pacientes |
| 11. La enseñanza de habilidades comunicativas afirma lo obvio y luego lo complica |
| 12. Aprender habilidades de comunicación es divertido |
| 13. deleted |
| 14. Aprender habilidades comunicativas me ha ayudado o me ayudará a respetar a mis compañeros |
| 15. Me resulta difícil confiar en la información sobre habilidades comunicativas que me dan los profesores de otras especialidades no clínicas. |
| 16. Aprender habilidades de comunicación me ha ayudado o me ayudará a reconocer los derechos de los pacientes en relación con la confidencialidad y el consentimiento informado. |
| 17. deleted |
| 18. Cuando solicité el ingreso en enfermero, pensé que era una buena idea aprender habilidades de comunicación. |
| 19. No necesito buenas habilidades de comunicación para ser enfermero |
| 20. Me cuesta admitir que tengo algunos problemas con mis habilidades comunicativas |
| 21. Creo que es muy útil aprender habilidades de comunicación en la carrera de enfermería |
| 22. deleted |
| 23. Aprender habilidades de comunicación es aplicable al aprendizaje de la enfermería |
| 24. Me cuesta tomarme en serio el aprendizaje de habilidades comunicativas |
| 25. Aprender habilidades comunicativas es importante porque mi destreza para comunicarme es una habilidad para toda la vida |
| 26. El aprendizaje de habilidades comunicativas debería recaer en los estudiantes de psicología, no en los de enfermería |
